# Tell Me About Your Visit With the Lions: Eliciting Event Narratives to Examine Children’s Memory and Learning During Summer Camp at a Local Zoo

**DOI:** 10.3389/fpsyg.2021.657454

**Published:** 2021-07-08

**Authors:** Tida Kian, Puneet K. Parmar, Giulia F. Fabiano, Thanujeni Pathman

**Affiliations:** ^1^Memory Development Laboratory, Department of Psychology, York University, Toronto, ON, Canada; ^2^LaMarsh Centre for Child and Youth Research, York University, Toronto, ON, Canada

**Keywords:** memory development in children, autobiographical memory, episodic memory, narratives, STEM learning, informal learning environments, semantic memory

## Abstract

School-aged children often participate in school field trips, summer camps or visits at informal learning institutions like zoos and museums. However, relatively little is known about children’s memory and learning from these experiences, what types of event details and facts are retained, how retention varies across age, and whether different patterns are observed for different types of experiences. We aimed to answer these questions through a partnership with a local zoo. Four- to 10-year-old children (*N* = 122) participated in a weeklong summer camp, during which they engaged in dynamic events, including visits to zoo animals. On the last day of camp, we elicited autobiographical event narratives for two types of experiences: a child-selected animal event (visit to their favorite animal) and an experimenter-selected animal event. We coded event narratives for length and breadth using previously used autobiographical memory (AM) narrative coding schemes. In addition, we created a coding scheme to examine retention of semantic information (facts). We report the types of autobiographical event details and facts children recalled in their narratives, as well as age group differences that were found to vary depending on the type of information and type of event. Through this naturalistic, yet controlled, study we gain insights into how children remember and learn through hands-on activities and exploration in this engaging and dynamic environment. We discuss how our results provide novel information that can be used by informal learning institutions to promote children’s memory and retention of science facts.

## Introduction

Visits to informal learning institutions like science centers, museums and zoos are common experiences (see discussion Haden, 2010). Thousands of children visit such institutions each year, whether through school field trips, day trips with parents and caregivers, or as part of registered camps (e.g., summer camp). Not only do such visits create memorable experiences, but such visits can also supplement formal (i.e., classroom) learning (Hudson and Fivush, 1991; Birney, 1995; Falk and Dierking, 1997; DeMarie, 2001; Davidson et al., 2009; see also Cox-Peterson et al., 2003). Multiple memory systems are involved in experiences like a museum trip or a visit to the giraffe exhibit at a zoo. Our episodic memory system allows us to remember the details of events that occurred at a specific time and place in the past (Tulving, 1984). Such memories can be autobiographical, in that they are based on personally significant or self-relevant events (e.g., Nelson and Fivush, 2004; Palombo et al., 2013). A child stating that the giraffe was eating leaves when she visited its enclosure is an example of an episodic memory. Another type of memory, semantic memory, allows us to retrieve general world knowledge or memory of facts (Tulving, 1972). For example, a child stating that giraffes have dark-colored tongues is a semantic memory or fact. Together these memory systems allow us to convert our experiences into lasting memories. However, relatively few studies have examined children’s autobiographical and semantic memories about these personal experiences (e.g., Imuta et al., 2018). In the present study we examined 4- to 10-year-old children’s memory and learning after a week-long summer camp at a local zoo by examining their event narratives.

Much of our understanding of autobiographical memory (AM) and its development is based on examination of autobiographical event narratives (for reviews see Nelson and Fivush, 2004; Pasupathi et al., 2007; Fivush, 2011; Haden and Hoffman, 2013; Bauer, 2015; Habermas and Reese, 2015). Narratives can be elicited via interviews in which an experimenter asks a participant to describe a past event with an open-ended question (e.g., “Tell me about the time you [event]”), followed-up with more specific *Wh-*questions (what, when, where, who, why and how; example “who was there when you [event]”) (see Haden and Hoffman, 2013). These questions allow a thorough description of the event in addition to participants’ thoughts, motivations and emotions related to the event (Nelson and Fivush, 2004). Researchers can then code and analyze narratives to further our understanding of changes across childhood.

This research shows that AM and the ability to narrate a past event emerges during the preschool years (Nelson and Fivush, 2004; Fivush, 2014). Between 16 and 18 months of age, infants can speak two-word utterances, and show some evidence of talk about past experiences (see Bloom, 1991, and discussion by Fivush, 2011). In early conversations, adults provide much of the structure and support of the content, however, children’s ability to provide narratives of their early experiences improves dramatically over subsequent years (Ornstein and Haden, 2001; Reese, 2002, 2014). With age, narratives begin to demonstrate organization through the inclusion of temporal and causal connections that allow children to link different actions together (Van den Broek, 1997). By 3.5 years, children become more capable of providing fairly coherent narratives of past experiences even after relatively long delays with slight prompting from adults (Fivush et al., 1987, 1995; Reese, 2002). Narrative skills continue to develop, showing increasing complexity and elaboration, throughout the preschool years into middle childhood (Haden et al., 1997) and afterward. In a 3-year longitudinal study, Bauer and Larkina (2019) reported steady increases in AM development across the age range of 4–10 years old. Researchers elicited narratives from 4-, 6-, and 8-year-old children about events that parents noted occurred within the past 4 months, and included events like celebrations, family outings and after-school events. With increasing age, children provided longer narratives (measured by the number of propositional units within the narrative). In addition, researchers found that with increasing age, narratives became more complete, as measured by narrative breadth (the number of different event detail or narrative categories mentioned, like who, what, when, etc.; see Bauer and Larkina, 2014, 2019). Further, past research shows that certain event details are more common in children’s event narratives than others. For example, in a study with a group of 7- to 10-year-olds, researchers found that the what-action (reference to actions or activities) and what-object (reference to objects present) narrative categories were most often present in children’s event narratives, whereas the why narrative category (reference to causation or justification) was least often present, for both recent and distant events (Bauer et al., 2007). Overall, the examination of children’s narratives about past events has been fruitful in furthering our understanding of what types of event details children provide, and in showing age-related improvements in AM across childhood (Reese, 1999; Bauer, 2007; Habermas and de Silveira, 2008; Pasupathi and Wainryb, 2010; Fivush, 2011; see also Peterson and McCabe, 1994; Wang and Leichtman, 2000; Hoerl, 2007).

Semantic memory and knowledge in childhood has been tested and discussed in various ways (e.g., see Chi, 1978; Nelson et al., 1983; Bjorklund, 1985; Mareschal and French, 2000). However, relatively few studies have examined both episodic or AM along with semantic memory measures in childhood (e.g., Robertson and Köhler, 2007; Sipe and Pathman, 2020) and few have examined the different types of semantic knowledge reported in AM narratives or interviews. Those that have seem to focus on one of two aspects of semantic memory. In one line of work, researchers have examined children’s semantic knowledge in terms of scripts or schemas for familiar events (school day, class trips; see also DeMarie et al., 2000). For example, Fivush et al. (1984) demonstrated that 5-year-old children were capable of retrieving both general information about a familiar experience (what happens during a class trip) and details about a specific episode of a novel experience (what happened during a particular class trip). In another line of work, researchers have examined personal semantic information provided in narratives or interviews. For example, Piolino et al. (2007) interviewed 7- to 13-year-olds about both personal semantic information (e.g., home address, names of childhood friends) and episodic information (describing particular events) from past time periods (e.g., current school year and last school year). They found age-related increases in their episodic memory measure, but not for their personal semantic information measure. In contrast, Picard et al. (2009) found that age was positively correlated with both the amount of episodic and personal semantic information provided by 6- to 11-year-olds. Willoughby et al. (2012) also found a similar pattern with 8- to 16-year-old children. Thus, the examination of autobiographical interviews in these studies have shown that with increasing age, children provide more episodic details. In terms of a type of semantic memory – personal semantic details – these studies have shown both age invariance and age-related increases. The purpose of the present paper was not to examine semantic memory in terms of schemas or event representations, nor examine semantic memory in terms of personal facts (e.g., “I am 9 years old”). Instead, one of our goals was to examine children’s inclusion of science-related and animal-related facts in their AM narratives based on particular experiences at a local zoo.

Zoos, and other informal learning institutions like museums and science centers, are ideal settings to study children’s memory and learning. Not only do such settings allow developmental scientists to study phenomena in the “messiness of the real world” (Golinkoff et al., 2017, p. 1407), but these settings have several features that are advantageous. First, unlike in lab-based studies in which children both experience and are tested on events within the lab, children can experience events in informal learning environments without knowing they will later be tested on those events. Further the events themselves are engaging and dynamic, especially compared to lab-based stimuli like pictures on a computer screen or even staged lab events. Since there are limits to ecological validity in lab-based settings (Schmuckler, 2001), naturalistic settings like zoos are a more representative place to test memory as a natural phenomenon. Second, visits to informal learning institutions are invaluable experiences because children can more directly interact with or see the objects they are learning about (see Davidson et al., 2009). Third, zoos, museums, and related institutions allow for social interactions among peers and educators which can be different than the social interactions during classroom learning; these social interactions have positive implications for learning (Birney, 1995; Davidson et al., 2009). Last, during visits to zoos, children can be given freedom to explore various exhibits or portions of exhibits, and thus be a source of free choice learning (Tofield et al., 2003). Further, qualitative research suggests that high personal involvement is one factor that makes museum experiences more memorable (Wolins et al., 1992).

Personal involvement or self-relevance has also been shown to affect the developmental trajectory of children’s memory performance. In a study by Pathman et al. (2011), children and adults took photographs during a museum visit; they were asked to reflect on how they felt about the object or exhibit they were photographing (*high* self-relevance; AM condition). Participants also answered questions about photographs of the same objects or exhibits taken by someone else, shown on a laptop at the museum (*low* self-relevance; episodic condition: mimicked lab-based studies). The participants were then invited to the laboratory 1–2 days after they visited the museum to test recognition of the photographs they had taken (autobiographical condition) and photographs they had viewed on the laptop (episodic condition). All age groups had higher levels of correct recognition in the autobiographical relative to the episodic condition. Importantly, younger children (7–9 years old) were less accurate than older children (9–11 years old) in the episodic condition, but these groups did not differ in the autobiographical condition. This work suggests that self-involvement and self-relevance can boost children’s memory and minimize age-related differences (Pathman et al., 2011) and parallels a robust literature in both adults and children showing better memory for words or events related to the self (e.g., Zhu et al., 2012; Cunningham et al., 2014; see Symons and Johnson, 1997, for review).

In the present study, we elicited memory narratives from 4- to 5-year-old, 6- to 7-year-old, and 8- to 10-year-old children about two particular events from a weeklong summer camp experience at a local zoo. Children described what they remember and learned from a visit to their favorite animal (child-selected event) and from a visit selected by the experimenter (experimenter-selected event). We then analyzed children’s memory narratives to examine the types of event details they included in their event narratives (both using a traditional AM coding scheme and a semantic fact coding scheme we created). We examined whether there were age-related differences in their narratives (narrative length, narrative breadth, and number of animal-related facts). Further, although we do not directly compare the child-selected and experimenter-selected event narratives, we explore whether patterns were similar or different across event types. We predict the autobiographical coding to show age-related improvements in both narrative length and breadth, paralleling past research. We cannot make strong predictions about the number or types of semantic facts recalled, given the novelty of this work, but expect older children to provide more facts in their narratives given improvements in language and ability to integrate events to produce new semantic knowledge (e.g., Bauer and Larkina, 2017; Varga et al., 2019). Different patterns of results for the child-selected compared to experimenter-selected event narratives could be expected given the literature on the importance of self-relevance in the memory literature and recommendations for museum educators (see Wolins et al., 1992).

## Materials and Methods

### Participants

Participants were 4- to 10-year-old children who took part in a 5-day summer camp at the Toronto Zoo that occurred during the months of July and August. Parents who had registered their children for camp received an email from the zoo advertising the study and were asked if they wanted their child to participate. The parental consent form was submitted online, and children gave verbal assent the day of the interview (Friday; see procedure below). The interview included two open-ended questions regarding the child’s experience visiting exhibits and animals while at zoo camp: child-selected event and experimenter-selected event (see procedure below). Only participants who had time to be asked at least one of the event questions are included in this study. Specifically, participants in this study were 122 children: 36 4- to 5-year-olds (*M*_age_ = 62.84 months, *SD* = 6.26; 20 girls, 16 boys), 39 6- to 7-year-olds (*M*_age_ = 84.03, *SD* = 7.16; 24 girls, 15 boys), and 47 8- to 10-year-olds (*M*_age_ = 108.5 months, *SD* = 8.70; 25 girls, 22 boys).

Demographics completed by parents online revealed that 55.74% percent of the children were Caucasian or White, 21.31% were Asian, 15.58% were mixed race, 3.28% were Latin American, 0.82% were Aboriginal/First Nations, and 0.82% were Black or African American/Canadian. An additional 2.46% of parents chose not to specify their child’s ethnicity. Most of our sample reported family income (before taxes; Canadian funds) greater than $90,000. The specific percentages are as follows: 52.46% reported family income greater than $120,000, 25.49% reported family income as $90,000–120,000, 9.84% reported family income as $60,000–90,000, 3.28% reported family income as $40,000–60,000, 1.64% reported family income as $25,000–40,000, and 0.82% reported family income as less than $15,000. An additional 7.38% of parents chose not to specify family income.

From the total of 122 participants, 102 of the participants have data for both the child-selected (favorite animal) and experimenter-selected animal question. Eighteen participants have data for the child-selected event question, but not the experimenter-selected event question. This was due to time constraints (*n* = 13), the child was absent on the day the experimenter-selected animal was visited (*n* = 1), they were not able to remember the event or animal visited (*n* = 1), or the child did not talk about the animal the experimenter selected (*n* = 3). Two additional children answered both questions, but their child-selected response was not included in the analysis. This was because the child-selected animal was not at the zoo (*n* = 1) or because the child discussed multiple favorite animals so we could not target their narrative to one event (*n* = 1). In sum, data analysis includes 120 children for the child-selected event (favorite animal) question, and 104 children for the experimented-selected event.

The protocol was reviewed by the York University Research Ethics Board. Children received a “Junior Scientist” certificate and parents were entered into a draw for a free 1-year membership or membership renewal to the Toronto Zoo.

### Procedure

Children took part in a 5-day summer zoo camp (Monday–Friday; full-day) that included various fun activities, crafts, games and interactions with zoo staff. Importantly, the camp also included visits to animals exhibits. During these visits children heard information about animals from camp counselors, zookeepers and staff. The schedules of which animal exhibits would be visited at which particular times during the week was pre-determined by zoo staff and provided to experimenters. Schedules varied per week depending on the theme and camp group. The schedules listed the specific areas of the zoo that would be visited each day, and experimenters knew which animals were located in each area of the zoo. Thus, if a child mentioned that they visited a giraffe, we could confirm this by checking whether this child’s schedule included a visit to the area of the zoo where the giraffe was located. In addition to the schedule listing the particular areas of the zoo the camp groups visited on a particular day, the schedule also listed specific animals visited during zookeeper talks (“Meet the Keeper,” “Behind the Scenes,” and “Enrichment”). These talks were scheduled at particular times of the day during which children saw the animal while they heard information about the animal from a zookeeper or zoo staff. Experimenters provided “camp counselor checklists” to each camp counselor so they could note any deviations from the pre-determined schedule each day (e.g., changes due to weather).

On a Friday, the last day of zoo camp, experimenters interviewed the children during planned “downtime” (e.g., after lunch), so as not to take children away from scheduled camp activities. The focus of the present work is on the elicitation of narratives about two events: a child-selected event (favorite animal) and an experimenter-selected event. For the first narrative obtained, the experimenter asked the participant to talk about the visit to their favorite animal. Since the child was able to select an animal of their choice, this event (hereafter referred to as the “child-selected event”) can be considered to have high self-relevance. For the second narrative obtained, the experimenter asked the participant to talk about a visit to an animal exhibit selected by the experimenter, based on the schedule (see below). Since the experimenter selected the event, this event (hereafter referred to as the “experimenter-selected event”) can be considered to have relatively low self-relevance. See Table 1 for the specific script and questions used to elicit the narratives. The experimenter asked free recall questions (e.g., “What do you remember …”) followed by WH-questions (e.g., “Who was there …”), adapted from previous AM narrative studies (e.g., Bauer and Larkina, 2019). In addition, the experimenter asked a question that was aimed at children’s fact knowledge (“What are some neat/cool things you learned about [animal]”). Questions/prompts for both events were similar but included some variations. Specifically, for the child-selected event, the experimenter asked the child the reason for selecting that animal as their favorite. However, this sub-question was not relevant for the experimenter-selected event.

**TABLE 1:** Autobiographical narratives elicitation script (interview questions).

Child-selected event	Experimented-selected event
You saw lots of different animals at the zoo this week. What was your favorite animal you saw at the zoo this week?	Your camp counselor took you to see an animal called the [animal name]. You got to see the [animal] while a keeper taught you all about it

• Why was it your favorite animal? • What do you remember about the time you saw the [name of animal] this week? • Who else was there? • What did you do when you were there? • What was the [animal] doing when you were there? • Where were you? • When was this? • How did you feel when you saw the [name of animal]? • What are some neat things you learned about the [name of animal]?	• What do you remember about the time you saw the keeper and the [animal]? • Who was near you when you saw the [animal]? • What did you do when you were there? • What was the [animal] doing when you were there? • Where were you? • When was this? • How did you feel when you were there? • What are some cool things you learned about the [name of animal]?

The event that would be used for the experimenter-selected event narrative was selected prior to testing. This event always included a scheduled talk about an animal (the local zoo’s names for these talks were: “Meet the Keeper,” “Behind the Scenes,” and “Enrichment” talks). During these talks the child visited the animal exhibit and a zoo staff or zookeeper presented information about the animal. Scheduled talks were used for this event narrative because these events were the only ones for which we could guarantee the child visited and saw the animal selected. For other parts of the schedule, only the exhibit location was mentioned so we could not be certain that an animal from an exhibit area was seen by a particular child. Thus, randomly selecting a scheduled talk from the child’s schedule ensured the child visited that animal.

The interviews were audio-recorded using a portable recorder. In addition, the experimenter wrote down responses on a data profile sheet as the child was speaking.

### Coding and Reliability

Audio recordings were transcribed verbatim and then reviewed by a second transcriber for accuracy. Whenever the participant’s voice was faint in the recording due to background noise, transcribers referred to the data profile sheet to fill in any gaps. Participants had two types of ID numbers: a participant ID created prior to the interview for identification purposes which was based on age group and a coding ID that was used while coding narratives to minimize coder bias. This coding ID was generated for each participant at random and gave no indication to the age group of the participant. The goal for reliability procedures was to have at least 20% of narratives coded by a reliability coder following guidelines and past papers (see Haden and Hoffman, 2013); precise percentages for the final data set are below for each type of coding system. The intraclass correlation coefficients reported for reliability analysis throughout this paper meet or exceed recommendations by Haden and Hoffman (2013).

#### Narrative Length

Propositional coding was conducted such that “one individual parsed all on-task contributions into propositional units (i.e., unit of meaning that included subject-verb construction)” (Bauer and Larkina, 2019, p. 66). That is, propositional coding was centered around verbs or verb phrases. For example, a child could say “We watched the lemurs eat” which would be parsed as [We watched] [the lemurs eat] (2 propositional units). To account for repeated information by children, we counted *unique* propositional units which contained unique and non-repeated information. These unique propositional units were summed to come up with a total number of propositional units, and provided us with what we will hereafter call “narrative length.” One primary coder coded all the transcripts and a reliability coder coded approximately 25% of transcripts for each of the two narratives (child-selected and experimenter-selected events). Intraclass correlation coefficients for child-selected and experimenter-selected events were 0.92 and 0.96, respectively. The primary coders’ judgments were used in all analyses.

#### Autobiographical Memory Coding and Narrative Breadth

We used the extensive coding manual developed by Bauer and colleagues (e.g., Bauer and Larkina, 2014; Bauer et al., 2017) to quantify the autobiographical event details children included in their narratives. This coding scheme is described in detail in previous studies (e.g., Bauer et al., 2007) and referred to as “narrative coding” (to describe the coding scheme) and “narrative categories” to refer to the individual codes. Given that our study involves additional coding of the narratives (i.e., semantic coding) we will refer to this as “AM coding” and “AM categories” instead.

We adapted the previously used AM coding category “WHAT-OBJ” to add a “WHAT-OBJ-A” category for mention of an animal or animal name. See Table 2 for explanation of the individual AM categories. For example, the sentence “The three cheetahs were sleeping” would receive the codes [HOW-DESC], [WHAT-OBJ-A], [WHAT-ACT]. Repeated information was not coded, such as repeat mentions of an animal name, unless it provided additional or novel detail. For each participant the number of codes in each particular AM code category was summed.

**TABLE 2:** Autobiographical memory coding scheme for narrative breadth (children’s descriptions about events).

Narrative category (AM codes)	When code was applied
Who	Specific mentions of people, gender, or a class of people present for or participating in the event (e.g., “Tim” and “camp counselor”)
What-object	Specific objects or things present in the event or activity being described (e.g., “soccer ball”)
What-object-animal*	The mention of an animal or specific name of an animal (e.g., “tiger”)
What-action	Actions or activities performed by a character or an object in the narrative (e.g., “jump”)
Where	Location of the event in place; a place/location that a person or object can go to (e.g., “in,” “on top of,” and “grandma’s house”)
When	Reference to time or placing the event in time, including indications of order of events within an experience (e.g., “yesterday” and “Tuesday”). Note this “when” category was split into a new coding scheme created by our lab which included individual sub-codes for “when,” but we summed sub-codes to create the “when” category for the present study to parallel past research
Why	Justification or causation statements illustrating the dependency of different aspects of the event (e.g., “because” and “until”)
How-description	Adverbs, adjectives, words, or prepositional phrases that describe the observable characteristics of an object or an action, such as length, height, number, color, and texture. This observation is without any personal evaluation (e.g., “it was pink”)
How-evaluation	A personal evaluation of the event, for example, through the use of an intensifier (e.g., “largest”), the use of a subjective modifier (e.g., “it was pretty”), or mention of an internal state (such as a term conveying information about emotion, cognition, perception, or physiological states) (e.g., “I am happy”)

*Code created for present study.

For AM coding, two primary coders coded 50% of the documents each that were in equal amount for gender and age group. To assess reliability, a third coder independently coded a randomly selected 27% of transcripts for the child-selected narratives and 31% for experimenter-selected narratives (transcripts were randomly selected; roughly proportional to the presentation of age groups; to meet goal that at least 20% of each primary coders’ transcripts would be coded by the independent reliability coder). This split of 50% of transcripts per primary coder has also been similarly employed in previous research (see Bauer and Larkina, 2016; Bauer et al., 2019). Intraclass correlation coefficient for the child-selected event was 0.99 for the total sum of all AM codes (individual AM categories intraclass correlation coefficients ranged from 0.91 to 0.99). Intraclass correlation coefficient for the experimenter-selected event was 0.99 for the total sum of all AM codes (individual AM categories intraclass correlation coefficients ranged from 0.90 to 0.99). The primary coders’ judgments were used in all analyses.

A narrative breadth score was calculated and used as a way to assess narrative completeness following past studies (e.g., Bauer et al., 2007; Bauer and Larkina, 2019). Specifically, AM codes were divided into 8 different categories used in past work (all categories from Table 2; note that What-Obj and What-Obj-A were considered 1 category for these purposes). For each event, children received one point for a code reflective of the category, regardless of the numbers of codes provided (max narrative breadth score = 8).

#### Semantic Coding (Animal Related Facts)

To assess semantic memory, scientific facts about the animals or related to the animals were coded for both the child-selected event and experimenter-selected event. Every meaningful unit or phrase that conveyed new information was coded (e.g., Benjamin et al., 2010; Imuta et al., 2018). Coding was based on different mutually exclusive categories and included the following: behavior fact (BF), targeting fact (TF), abstract fact (AF), concrete fact (CF), and evaluative fact (EF). See Table 3 for examples of all semantic fact codes. A BF is reference to animal movement or action or any habits which may or may not be seen at the time of zoo visit. A TF is given when a child mentions a specific type of animal (“spider monkey” or “golden lion monkey”) or subgroup of animal (“baby monkey” or “female monkey”). However, a TF code is not awarded when a child refers to a general term “monkey.” Participants received credit for an AF if they referred to any unobservable scientific information at the time of zoo visit. AFs could include information about the animal or information directly related to the animal (e.g., habitat). Any information about the physical appearance of the animal, animal-relevant objects or surroundings that is directly observable was coded as a CF. Any description, explanation or information about the animal that could be considered an evaluation based on facts or what the child may know about the animal received the code EF. Multiple examples are provided in Table 3 for each semantic code category.

**TABLE 3:** Semantic coding scheme.

Type of code	Definition	Examples
Behavior fact (BF)	Any information referring to animal movement or action or any habits which may or may not have been seen at the time of zoo visit	Tigers are good *climbers*Male bats *fly* quickly
Targeting fact (TF)	Mention of the specific type/kind of animal or subgroup of animal. Not just a label of an animal but requires narrowing or targeting to a more specific animal or animal category.	*Amur tigers* are going extinct*Spider monkeys* are clever
		*Male* deer have antlers
Abstract fact (AF)	Any scientific information about the animal or related to the animal which was unobservable at the time of zoo visit	Rainforests are *warm*Elephants *are hunted for* their tusks
Concrete fact (CF)	Any fact related to the physical appearance of animal (or animal-relevant objects or surroundings) that was directly observable at the time of zoo visit	Giraffes have *dark tongues*The males use their *antlers* to fight
		Polar bears swim in cold *water*
Evaluative fact (EF)	Any description, explanation or information about the animal that could be considered an evaluation based on facts or what the child may know about the animal	Tortoises are *nice*Otters are *cute*

*The above italics highlight particular fact codes for that category. However, these sentences can contain multiple different semantic codes. Examples: Amur tigers (TF) are going extinct (AF). The *males* (TF) *use* (BF) their *antlers* (CF) to *fight* (BF). Elephants *are hunted for* (AF) their *tusks* (CF). Polar bears swim (BF) in cold (AF) water (CF).*

One point was assigned for each code and summed for each of these coding categories. Then all codes across all categories were summed to create an “overall semantic score.” A unit of information could not receive more than one of these codes. For example, if participant stated, “Zebras stay together so that their stripes are confusing to the other animals” they received three codes including [*stay together*] (BF), [*stripes*] (CF), and [*confusing to the other animals*] (AF).

The entire narrative (i.e., all on-topic talk in response to the questions asked in Table 1) was coded for both AM coding (described in previous sub-section) and semantic coding. For AM coding, we did not focus on particular sentences or sentence tense paralleling past AM narrative studies that have used this AM coding scheme (e.g., Bauer and Larkina, 2014). However, only certain sentences were considered facts for semantic coding. Specifically, information that was generalized or given in the present tense were considered facts; only such sentences received semantic codes. This coding scheme is consistent with the scientific information category in Imuta et al. (2018). In our study, “Cheetahs run fast” would receive the following AM codes for *Cheetahs* [WHAT-OBJ-A] *run* [WHAT-ACT] and *fast* [HOW-DESC]. This sentence would also receive semantic codes for Cheetahs *run* [BF] *fast* [AF]. However, a sentence like “the cheetah was yellow” would receive AM codes, but not semantic codes, since it referred to a specific episodic memory and not generalized semantic knowledge. In addition, only semantic details that were plausible were given semantic codes; if a child had said “Cheetahs are purple and green,” that would not have received semantic codes.

Our identification of the sentences that would and would not receive semantic codes shares some parallels to previous research about conceptual development that distinguish “generic” statements (statements about a kind of category) and “non-generic” or “specific” statements (statements about a particular member of a category; statements about a particular point in time) (e.g., Brandone and Gelman, 2009; Rhodes et al., 2012; Gelman et al., 2013; Foster-Hanson et al., 2016). In our coding scheme, statements considered “generic” by Gelman and colleagues would be coded as semantic facts in our study if they were in present tense. Sentences in past tense that referred to a specific point in time would be coded as “non-generic” by Brandone and Gelman (2009); such a sentence would not receive semantic codes in our study. However, some “non-generic” statements in present tense would receive semantic codes in our study. For example, “*some* cougars can run faster than others” would be considered “non-generic” (e.g., Gelman et al., 2013) because it does not refer to the entire category of cougars; this statement would be given semantic codes in our study.

One primary coder coded all the transcripts and a reliability coder coded 23% of transcripts for each of the two narratives (child-selected and experimenter-selected events). The intraclass correlation coefficient for the child-selected event was 0.98 for the overall semantic score (individual semantic code intraclass correlation coefficients ranged from 0.90 to 0.96). Intraclass correlation coefficient for the experimenter-selected event was 0.96 for the overall semantic score (individual semantic code intraclass correlation coefficients ranged from 0.85 to 0.96). The primary coders’ judgments were used in all analyses.

##### Semantic Propositional Units

In order to determine the amount of talk that was “semantic-related talk” we counted the unique propositional units that were from sentences that were in the present tense and seemed to convey generalized knowledge. For example, the phrase [Cougars run fast] conveys a scientific animal-related fact and was counted as a semantic propositional unit, but [the cougar was lying down] was considered an episodic description of what the cougar was doing during the time the child was observing it and as such was not considered a unique semantic proposition. Unique semantic propositions were summed for each participant. Percentages of the sum of unique semantic propositions (e.g., sentences like “cougars run fast”) relative to the overall number of unique propositional units (i.e., “narrative length”) were calculated. These values are used in analyses to assess whether there are age-related differences in the percentage of fact-like statements provided in the entire unique, non-repeated narrative, for both the child-selected and experimenter-selected events.

## Results

We report the number and types of details provided by children for each event narrative (child-selected event and experimenter-selected event), and whether there were age group differences in narrative length and breadth (completeness of narratives), and whether there were age group differences in the semantic information provided by children. Analyses were planned to be conducted for each event narrative separately, since various known methodological differences prevent us from directly comparing values obtained for these two narratives (e.g., we knew that not all children would have time to complete both narratives, and thus child-selected event was always discussed before experimenter-selected event; experimented-selected event were associated with keeper talks, which was not always the case for child-selected events).

### Child-Selected Event Narrative (Favorite Animal)

#### Preliminary Analyses

##### Delay

We conducted preliminary analyses to determine whether age groups varied in the delay between each child’s favorite animal visit and the testing session. Delay was determined by examining individual camp schedules and camp counselor checklists and noting what day of the week the child-selected animal was visited. For example, if a child selected the cheetah visit for this event narrative, and they visited the cheetah on Tuesday, then this child’s delay would be 3 days. Precise delay information was not available for 14 children because a child’s report of their favorite animal visit (e.g., “rhino”) did not allow us to isolate the visit to one particular location/time on the child’s schedule (e.g., both the Indian Rhino and White Rhino were visited on different days).

An analysis of variance (ANOVA) found no main effect of age group on delay, *F*(2,103) = 0.11, *p* = 0.90, $\eta_{\text{p}}^{2}$ = 0.002. Thus, the delay between the event and the testing session did not vary by age group (mean delay was between 2.00 and 2.14 days for each age group).

##### Gender

To determine whether gender differences influenced memory narratives, we conducted the analyses reported below (narrative length, narrative breadth, and semantic coding) with both age group and gender as factors. No significant main effects or interactions with gender were found (all *p*s > 0.05), and thus gender is not considered further for this event narrative.

#### Narrative Length

As a reminder, narrative length refers to the number of propositional units produced by the child across the entire narrative (unique information, not repeated; on-task contributions). The ANOVA indicated a main effect of age group, *F*(2,117) = 12.98, *p* < 0.001, $\eta_{\text{p}}^{2}$ = 0.18. Pairwise comparisons showed that narratives produced by 8- to 10-year-olds (*M* = 19.91, SD = 7.28) were longer than both 4- to 5-year-old (*M* = 11.71, SD = 8.25) and 6- to 7-year-old (*M* = 14.58, SD = 6.83) children (*p*s < 0.01). The length of narrative between the two youngest age groups did not differ (*p* = 0.10).

We found significant differences in narrative length, paralleling previous papers. Thus, subsequent analyses are reported both without and with narrative length controlled. Paralleling the rationale used by Bauer and colleagues (2017), we report both because “each permits a valid – and unique – perspective on the data” (p. 419). Not controlling for narrative length allows us to determine the number and types of AM and semantic codes that are naturally recalled in the narrative; after all, providing a coherent and complete narrative requires words. At the same time, controlling narrative length allows us to determine potential age group differences above and beyond variance explained by talkativeness. Our measure of narrative length involves unique, not repeated, talk. Still, one could argue that any age differences in the amount of AM or semantic codes could be explained by differences in how long individuals speak, since this increases opportunities to showcase particular AM or semantic codes. Thus, like past research (Bauer et al., 2017) we describe both ANOVA and ANCOVA (analysis of covariance) findings.

#### Autobiographical Memory Coding and Narrative Breadth

Descriptive statistics for the sum of individual AM code categories for each age group are reported in Table 4. Table 4 also includes analyses examining whether there are age group differences for each AM code category.

**TABLE 4:** Descriptive statistics for each AM code for the child-selected event.

Narrative category	Age groups					
	Overall	4- to 5-year-olds	6- to 7-year-olds	8- to 10-year-olds	Age-group differences
		*M* (SD)	*M* (SD)	*M* (SD)	*M* (SD)
Sum of narrative codes	35.87 (22.60)	26.20 (16.97)	32.50 (21.18)	45.77 (23.82)	***F*(2, 117) = 9.26, *p* < 0.001,$\eta_{\text{p}}^{2}$ = 0.14**
					[4- to 5- and 6- to 7-year-olds < 8- to 10-year-olds]
Who	2.57 (2.51)	1.31 (1.02)	2.11 (1.69)	3.87 (3.17)	***F*(2, 117) = 13.85, *p* < 0.001,$\eta_{\text{p}}^{2}$ = 0.19**
					[4- to 5- and 6- to 7-year-olds < 8- to 10-year-olds]
What-object	2.93 (3.79)	2.49 (3.40)	2.29 (3.50)	3.79 (4.18)	*F*(2, 117) = 2.02, *p* = 0.14, $\eta_{\text{p}}^{2}$ = 0.03
What-object-animal	3.43 (2.79)	2.57 (2.02)	3.18 (2.93)	4.26 (2.98)	***F*(2, 117) = 4.06, *p* = 0.02,$\eta_{\text{p}}^{2}$ = 0.07**
					[4- to 5-year-olds < 8- to 10-year-olds]
What-action	9.45 (7.09)	6.94 (6.27)	8.42 (7.30)	12.15 (6.70)	***F*(2,117) = 6.56, *p* = 0.002,$\eta_{\text{p}}^{2}$ = 0.10**
					[4- to 5- and 6- to 7-year-olds < 8- to 10-year-olds]
Where	2.18 (2.28)	1.23 (1.28)	2.26 (2.40)	2.81 (2.57)	***F*(2, 117) = 5.18, *p* = 0.007,$\eta_{\text{p}}^{2}$ = 0.08**
					[4- to 5-year-olds < 6- to 7-year-olds and 8- to 10-year-olds]
When	3.26 (2.94)	1.51 (1.58)	3.63 (4.96)	4.25 (3.89)	***F*(2, 117) = 5.49, *p* = 0.005,$\eta_{\text{p}}^{2}$ = 0.09**
					[4- to 5-year-olds < 6- to 7-year-olds and 8- to 10-year-olds]
Why	1.42 (1.24)	1.29 (1.02)	1.24 (0.97)	1.66 (1.54)	*F*(2, 117) = 1.51, *p* = 0.23, $\eta_{\text{p}}^{2}$ = 0.03
How-description	3.58 (3.30)	2.31 (1.94)	2.76 (2.54)	5.17 (3.97)	***F*(2, 117) = 10.74, *p* < 0.001,$\eta_{\text{p}}^{2}$ = 0.16**
					[4- to 5- and 6- to 7-year-olds < 8- to 10-year-olds]
How-evaluation	4.04 (3.70)	2.60 (2.77)	3.71 (2.73)	5.38 (4.51)	***F*(2, 117) = 6.42, *p* = 0.002,$\eta_{\text{p}}^{2}$ = 0.10**
					[4- to 5- and 6- to 7-year-olds < 8- to 10-year-olds]

*For significant main effects of age (bolded statistics), pairwise comparison of age group differences that had a *p* < 0.05 are summarized in square brackets.*

To test for age-related differences in the breadth or completeness of children’s narratives an ANOVA was conducted with age group as a between-subjects factor. We found a main effect, *F*(2,117) = 5.90, *p* = 0.004, $\eta_{\text{p}}^{2}$ = 0.09, and pairwise comparisons revealed the following pattern: 4- to 5-year-olds (*M* = 6.71, SD = 1.51) had a lower narrative breadth score than both 6- to 7-year-olds (*M* = 7.21, SD = 0.88) and 8- to 10-year-olds (*M* = 7.53, SD = 0.78), *p*s < 0.05. The two oldest age groups did not differ in narrative breadth (*p* = 0.17).

The ANCOVA, with narrative length as a covariate, found no main effect of age group on narrative breadth score, *F*(2,116) = 1.11, *p* = 0.33, $\eta_{\text{p}}^{2}$ = 0.02. Thus, once narrative length was considered, age group differences in narrative breadth or completeness of narratives disappeared for the favorite animal event.

#### Semantic Coding (Animal Related Facts)

As a reminder, we coded children’s narratives for various types of semantic facts. Each participant received a score for the different semantic code categories (BF, TF, AF, EF, and CF), in addition to an overall score which summed values across all semantic code categories. Overall semantic score was assessed with an ANOVA, and then followed by an ANCOVA, controlling for narrative length, for reasons described earlier.

The ANOVA showed age-related improvements in the overall number of facts children recalled in their narratives *F*(2,117) = 6.55, *p* = 0.002, $\eta_{\text{p}}^{2}$ = 0.10. Pairwise comparisons revealed that 8- to 10-year-olds (*M* = 7.80, SD = 5.07) reported more facts than both 4- to 5-year-olds (*M* = 4.14, SD = 4.31) and 6- to 7-year-olds (*M* = 5.47, SD = 4.35), *p*s < 0.03. The two youngest groups did not differ (*p* = 0.22). The descriptive statistics for each age group and analyses of age differences for separate semantic codes are reported in Table 5. As can be seen in Table 5, age group differences in overall semantic score seems to be driven by age group differences in TF, AF, and CF codes.

**TABLE 5:** Types of recalled facts for child-selected event.

Type of fact	Age groups			
	4–5	6–7	8–10	Age-group differences
	*M* (*SD*)	*M* (*SD*)	*M* (*SD*)	
Behavior fact	1.09 (1.69)	1.18 (1.81)	1.66 (1.55)	*F*(2,117) = 1.42, *p* = 0.25, $\eta_{\text{p}}^{2}$ = 0.02
Targeting fact	0.54 (0.74)	0.79 (1.04)	1.11 (1.13)	***F*(2,117) = 3.25, *p* = 0.04,$\eta_{\text{p}}^{2}$ = 0.05**
				[4- to 5-year-olds < 8- to 10-year-olds]
Abstract fact	1.14 (1.60)	1.89 (2.00)	2.55 (2.56)	***F*(2,117) = 4.37, *p* = 0.02,$\eta_{\text{p}}^{2}$ = 0.07**
				[4- to 5-year-olds < 8- to 10-year-olds]
Concrete fact	1.03 (1.32)	1.08 (1.10)	1.81 (1.66)	***F*(2,117) = 4.13, *p* = 0.02,$\eta_{\text{p}}^{2}$ = 0.07**
				[4- to 5- and 6- to 7-year-olds < 8- to 10-year-olds]
Evaluative fact	0.34 (0.68)	0.47 (0.80)	0.66 (0.64)	*F*(2,117) = 2.10, *p* = 0.13, $\eta_{\text{p}}^{2}$ = 0.04

*For significant main effects of age (bolded statistics), pairwise comparison of age group differences that had a *p* < 0.05 are summarized in square brackets.*

The ANCOVA revealed no main effect of age group, *F*(2,116) = 0.09, *p* = 0.91, $\eta_{\text{p}}^{2}$ = 0.002. Thus, when narrative length was considered in the analysis, age group differences in the overall number of semantic facts provided was no longer apparent.

We also conducted an analysis to determine what percentage of the overall narrative length would be considered semantic-related talk (see section “Semantic Propositional Units”). As a reminder, we calculated the percentage of unique *semantic* propositional units (e.g., sentences like “Flamingos are pink” or “Amur tigers are endangered”) from the overall number of unique propositional units in the narrative (the value used in the “narrative length” score above). For the child-selected (favorite animal) event narrative there were no age-group differences in the percentage of the narrative that could be considered semantic talk, *F*(2,117) = 0.93, *p* = 0.40, $\eta_{\text{p}}^{2}$ = 0.02. Percentages for each age group were as follows: 4- to 5-year-olds (*M* = 32.14%, *SD* = 15.34), 6- to 7-year-olds (*M* = 28.60%, SD = 10.57), and 8- to 10-year-olds (*M* = 28.57%, SD = 12.75). Thus, although there were age group differences in the amount of overall talk, there were no age group differences in the amount of talk in which children made generalizations or fact-like statements.

#### *Post hoc* Analyses

A partial correlation, controlling for both age in months and narrative length, revealed no relation between narrative breadth score and overall semantic score, *r*(116) = 0.06, *p* = 0.53. (Note there is a positive correlation between these variables when narrative length is not included as a control variable).

### Experimenter-Selected Event

#### Preliminary Analysis

##### Delay

Similar to the child-selected question, we conducted a preliminary analysis to determine whether age groups varied in the delay between the time in which they visited the experimenter-selected event and the testing session. Delay was determined in the same manner as described in the child-selected event narrative delay analysis. All children who answered the experimenter-specified question were considered for this analysis as the exact time and day of this event was specified in the schedule.

The ANOVA found a main effect of age group on delay, *F*(2,100) = 12.70, *p* < 0.001, $\eta_{\text{p}}^{2}$ = 0.20. Pairwise comparisons revealed that 8- to 10-year-olds (*M* = 2.71, SD = 0.56) had a longer delay than 6- to 7-year-olds (*M* = 2.20, SD = 0.93) (*p* = 0.006), and 6- to 7-year-olds had a longer delay than 4- to 5-year-olds (*M* = 1.74, SD = 0.86) (*p* = 0.02). As time delay does differ with age group, later analysis will report values with and without time delay controlled. This will allow us to determine whether this significant interaction with time-delay later impacts narrative length, breadth, and semantic coding.

##### Gender

To determine whether gender differences influenced memory narratives, we conducted the analyses reported below (narrative length, narrative breadth, and semantic coding) with both age group and gender as factors. No significant main effects of gender or interactions with gender were found for narrative length nor narrative breadth (*p*s > 0.05). Thus, gender is not discussed further for narrative length or narrative breadth subsections below. For semantic coding, a significant interaction between age group and gender was found (*p*s < 0.05). Follow-up revealed the effects were due to gender differences for the 8- to 10-year-old age group in facts recalled. These results are reported in the semantic coding subsection below.

#### Narrative Length

An ANOVA conducted for narrative length indicated a main effect of age group, *F*(2,101) = 14.90, *p* < 0.001, $\eta_{\text{p}}^{2}$ = 0.23. Pairwise comparisons showed that narratives produced by 8- to 10-year-olds (*M* = 18.85, SD = 9.85) were longer than both 4- to 5-year-olds (*M* = 8.85, SD = 4.28) and 6- to 7-year-olds (*M* = 12.92, SD = 6.49) children (*p*s < 0.001). The length of narrative between the two youngest age groups also showed that 6- to 7-year-olds produced longer narratives than 4- to 5-year-olds (*p* = 0.04).

As a significant effect for narrative length was found for all age groups, further analyses will be reported both without (ANOVA) and with (ANCOVA) controlling for narrative length.

#### Autobiographical Memory Coding and Narrative Breadth

Descriptive statistics for individual AM codes for each age group are reported in Table 6. Analyses of age group differences for each AM code category are also provided in Table 6.

**TABLE 6:** Descriptive statistics for each AM code for the experimenter-selected event.

Narrative category	Age group				
	Overall	4–5	6–7	8–10	Age-group differences
		*M* (SD)	*M* (SD)	*M* (SD)	
Sum of narrative codes	31.86 (27.02)	19.11 (8.06)	28.94 (14.74)	42.80 (37.52)	***F*(2, 101) = 7.39, *p* = 0.001,$\eta_{\text{p}}^{2}$ = 0.13**
					[4- to 5- and 6- to 7-year-olds < 8- to 10-year-olds]
Who	2.38 (1.80)	1.67 (1.11)	2.22 (1.67)	2.98 (2.09)	***F*(2, 101) = 4.83, *p* = 0.01,$\eta_{\text{p}}^{2}$ = 0.09**
					[4- to 5-year-olds < 8- to 10-year-olds]
What-object	2.73 (3.49)	1.52 (1.58)	2.83 (3.96)	3.44 (3.80)	*F*(2, 101) = 2.57, *p* = 0.08, $\eta_{\text{p}}^{2}$ = 0.05
What-object-animal	3.24 (3.98)	1.81 (2.15)	2.86 (2.02)	4.51 (5.58)	***F*(2, 101) = 4.24, *p* = 0.02,$\eta_{\text{p}}^{2}$ = 0.08**
					[4- to 5-year-olds < 8- to 10-year-olds]
What-action	9.31 (9.13)	4.97 (2.47)	8.22 (5.04)	13.12 (12.64)	***F*(2, 101) = 7.80, *p* = 0.001,$\eta_{\text{p}}^{2}$ = 0*.1*3**
					[4- to 5- and 6- to 7-year-olds < 8- to 10-year-olds]
Where	1.99 (2.70)	0.89 (1.15)	1.64 (1.59)	3.02 (3.68)	***F*(2, 101) = 6.15, *p* = 0.003,$\eta_{\text{p}}^{2}$ = 0.11**
					[4- to 5- and 6- to 7-year-olds < 8- to 10-year-olds]
When	2.78 (4.47)	1.52 (1.45)	2.25 (1.86)	4.12 (6.64)	***F*(2, 101) = 3.30, *p* = 0.04,$\eta_{\text{p}}^{2}$ = 0.06**
					[4- to 5-year-olds < 8- to 10-year-olds]
Why	0.65 (1.85)	0.19 (0.48)	0.31 (0.89)	1.27 (2.70)	***F*(2, 101) = 4.00, *p* = 0.02,$\eta_{\text{p}}^{2}$ = 0.07**
					[4- to 5- and 6- to 7-year-olds < 8- to 10-year-olds]
How-description	3.06 (3.42)	1.74 (2.18)	2.83 (2.80)	4.12 (4.22)	***F*(2, 101) = 4.32, *p* = 0.02,$\eta_{\text{p}}^{2}$ = 0.08**
					[4- to 5-year-olds < 8- to 10-year-olds]
How-evaluation	2.81 (2.35)	1.30 (0.10)	2.78 (1.80)	3.83 (2.85)	***F*(2, 101) = 11.38, *p* < 0.001,$\eta_{\text{p}}^{2}$ = 0.18**
					[4- to 5-year-olds < 6- to 7-year-olds < 8- to 10-year-olds]

*For significant main effects of age (bolded statistics), pairwise comparison of age group differences that had a *p* < 0.05 are summarized in square brackets.*

To test for age-related differences in the breadth or completeness of children’s narratives, an ANOVA was conducted with age group as a between-subjects factor. We found a main effect, *F*(2,101) = 13.89, *p* < 0.001, $\eta_{\text{p}}^{2}$ = 0.22, and pairwise comparisons revealed the following pattern: 4- to 5-year-olds (*M* = 5.48, SD = 1.76) had a lower narrative breadth score than both 6- to 7-year-olds (*M* = 6.17, SD = 1.42) and 8- to 10-year-olds (*M* = 7.20, SD = 0.90); *p*s < 0.05; 8- to 10-year-olds’ narrative breadth scores were higher than 6- to 7-year-olds’ scores (*p* = 0.001).

An ANCOVA, controlling for time delay, revealed a main effect of age group for narrative breadth, *F*(2,99) = 14.14, *p* < 0.001, $\eta_{\text{p}}^{2}$ = 0.22. Pairwise comparisons revealed that, with time delay used as a covariate, the above pattern holds: 8- to 10-year-olds had a higher narrative breadth score than 6- to 7-year-olds, and 6- to 7-year-olds had a higher narrative breadth score than 4- to 5-year-olds, *p*s < 0.02.

The ANCOVA, controlling for narrative length, did not reach significance for age-related differences on narrative breadth score, *F*(2,100) = 2.63, *p* = 0.08, $\eta_{\text{p}}^{2}$ = 0.05. The ANCOVA, controlling for time delay and narrative length, showed a main effect of age group, *F*(2,98) = 3.38, *p* = 0.04, $\eta_{\text{p}}^{2}$ = 0.06. Pairwise comparisons revealed that 8- to 10-year-olds had a higher narrative breadth score than 4- to 5-year-olds (*p* = 0.01). The two younger groups did not differ from one another (*p* = 0.17). Similarly, the two oldest age groups did not differ from one another in their narrative breadth (*p* = 0.09). Thus, after accounting for time delay and narrative length, we continue to see age-related differences for narrative breadth.

#### Semantic Coding (Animal Related Facts)

Overall semantic score was assessed with an ANOVA, which was followed by ANCOVAs controlling for narrative length and time delay for reasons mentioned above. The ANOVA showed age-related improvements in the overall number of facts children recalled in their narratives *F*(2,101) = 11.48, *p* < 0.001, $\eta_{\text{p}}^{2}$ = 0.19. Pairwise comparisons revealed the narratives of 8- to 10-year-olds (*M* = 6.71, SD = 5.65) included more semantic facts than the narratives of 6- to 7-year-olds (*M* = 4.22, SD = 3.91), *p* < 0.001. Four to 5-year-olds (*M* = 1.59, SD = 1.76) included fewer semantic facts than both older age groups (*p*s < 0.02). The descriptive statistics for each age group as well as analysis of age difference for separate semantic codes are reported in Table 7. Age group differences in overall semantic codes seems to be driven by the age group differences in BF, TF, and AF codes.

**TABLE 7:** Types of recalled facts for experimented-selected event.

Type of fact	Age groups			
	4–5	6–7	8–10	Age-group differences
	*M* (SD)	*M* (SD)	*M* (SD)	
Behavior fact	0.37 (0.69)	1.11 (1.35)	1.80 (1.85)	***F*(2,101) = 8.06, *p* < 0.001,$\eta_{\text{p}}^{2}$ = 0.14**
				[4- to 5-year-olds < 6- to 7-year-olds < 8- to 10-year-olds]
Targeting fact	0.15 (0.36)	0.56 (1.54)	1.05 (1.55)	***F*(2,101) = 3.78, *p* = 0.03,$\eta_{\text{p}}^{2}$ = 0.07**
				[4- to 5-year-olds < 8- to 10-year-olds]
Abstract fact	0.56 (0.97)	1.78 (2.06)	2.71 (2.76)	***F*(2,101) = 7.99, *p* < 0.001,$\eta_{\text{p}}^{2}$ = 0.14**
				[4- to 5-year-olds < 6- to 7-year-olds and 8- to 10-year-olds]
Concrete fact	0.44 (0.93)	0.56 (1.08)	0.98 (1.80)	*F*(2,101) = 1.48, *p* = 0.23, $\eta_{\text{p}}^{2}$ = 0.03
Evaluative fact	0.07 (0.39)	0.22 (0.42)	0.15 (0.36)	*F*(2,101) = 1.14, *p* = 0.33, $\eta_{\text{p}}^{2}$ = 0.02

*For significant main effects of age (bolded statistics), pairwise comparison of age group differences that had a *p* < 0.05 are summarized in square brackets.*

With an ANCOVA controlling for time delay, a main effect of age group was found, *F*(2,99) = 9.41, *p* < 0.001, $\eta_{\text{p}}^{2}$ = 0.16. The above age group pattern holds: 4- to 5-year-olds had a lower overall number of semantic codes compared with 6- to 7-year-olds and 8- to 10-year-olds, *p*s < 0.02; 6- to 7-year-olds had a lower overall number of semantic codes than 8- to 10-year-olds (*p* = 0.02).

With an ANCOVA controlling for narrative length, there was no main effect of age group in the overall semantic score, *F*(2,100) = 1.19, *p* = 0.31, $\eta_{\text{p}}^{2}$ = 0.02. When we conduct an ANCOVA controlling for both narrative length and time delay, there were no main effect for age group in the overall semantic score, *F*(2,98) = 0.94, *p* = 0.39, $\eta_{\text{p}}^{2}$ = 0.02. Thus, when controlling for narrative length and time delay, we do not find significant age-related differences in overall number of facts provided.

For the experimenter-selected event narrative the percentage of semantic-related talk for each age group were as follows: 4- to 5-year-olds (*M* = 9.48%, SD = 10.38), 6- to 7-year-olds (*M* = 18.33%, SD = 16.48), and 8- to 10-year-olds (*M* = 19.96%, SD = 11.64). Not only did we find age group differences in the amount of overall talk, but we also found age group differences in the amount of talk in which children made generalizations or fact-like statements, *F*(2,101) = 5.52, *p* = 0.005, $\eta_{\text{p}}^{2}$ = 0.10. Pairwise comparisons revealed 4- to 5-year-olds’ percentage of semantic talk was lower than that for both older age groups (*p*s < 0.02). Six to 7-year-olds and 8- to 10-year-olds did not perform differently (*p*s = 0.59). Thus, there were age-related differences in the percentage of fact-like statements children included in their narratives for the experimenter-selected event.

##### Gender

A significant interaction between age group and gender was found when gender was included as a factor in the above analyses. For example, for overall semantic score the ANOVA for this experimenter-selected event narrative revealed an interaction between age group and gender, *F*(2, 98) = 4.37, *p* = 0.02, $\eta_{\text{p}}^{2}$ = 0.08. To follow-up this analysis, we conducted analyses for each age group separately and found that for the 8- to 10-year-old group only, boys (*M* = 9.11, SD = 6.18) included more semantic facts in their narratives than girls (*M* = 4.64, SD = 4.29), *t*(39) = 2.72, *p* = 0.01. There were no gender differences for the two youngest age groups, *t*s < 0.5, *p*s > 0.62. To check whether the gender difference for the 8- to 10-year-olds could be due to a difference between boys and girls in the delay between experience of the experimenter-selected event and test, we conducted a *t-*test but found no difference between boys and girls in delay, *t* = 0.31, *p* = 0.76. To determine whether outliers could explain these results, we removed 2 boys whose overall semantic score was greater than 18 (mean score for age group + 2 × SD for age group). Even with these outliers removed from the analysis, the gender difference for overall semantic score remained: *t*(37) = 2.12, *p* = 0.04.

#### *Post hoc* Analyses

A partial correlation, controlling for both age in months and narrative length, revealed no relation between narrative breadth score and overall semantic score, *r*(100) = 0.05, *p* = 0.61. (Note there is a positive correlation between these variables when narrative length is not included as a control variable).

### Descriptive Comparison of Both Events

Overall children provided relatively long narratives, and anecdotally, children in our study were excited to talk about their experiences visiting the animal exhibits at this local zoo. We do not directly compare the child-selected and experimenter-selected narratives in analyses for the reasons described previously. However, we did plan to compare patterns found on an exploratory basis and report this here.

For both the child-selected and experimenter-selected event narratives, we found age-related increases in narrative length. For the child-selected event, 8- to 10-year-olds provided longer narratives compared to the two youngest age groups, which did not differ. For the experimenter-selected event, all three age groups were different from each other and showed steady increases. We note the different age patterns for the child-selected event (favorite animal) compared to experimenter-selected event narratives. Specifically, the 4- to 5- and 6- to 7-year-old groups did not differ in narrative length for the child-selected event, but 6- to 7-year-olds provided longer narratives than 4- to 5-year-olds for the experimenter-selected event.

For the child-selected event narrative, we found that the two oldest age groups (which did not differ from each other) had a higher narrative breadth score than the youngest age group. When we controlled for narrative length, however, the age-related differences in narrative breadth for the child-selected event narrative disappeared. For the experimenter-selected event narrative, all three age groups differed with age-related improvements throughout this period of childhood (when we did not control for narrative length). When we did control for relevant factors (delay, narrative length, both delay and length) we found that age group differences remained.

In terms of the semantic facts included in narratives, we found that for the child-selected event, 8-year-olds’ narratives contained a higher number of facts (overall semantic score) compared to both younger age groups, which did not differ from each other. However, when we controlled for narrative length the age groups no longer differed in the number of overall facts for the child-selected event narrative. For the experimenter-selected animal, we found that all 3 age groups differed from each other and showed steady increases in the number of facts provided in this 4- to 10-year-old age range. However, when we controlled for narrative length, these age-group differences disappeared.

## Discussion

The purpose of the present research was to examine children’s memory and learning from a week-long experience at a local zoo. Our primary goals were to examine 4- to 10-year-olds’ autobiographical event narratives to determine what types of event details and facts are recalled in narratives and how narratives differ between age groups. We also examined whether there would be relations between individual differences in event details and facts (i.e., are the children who included more types of AM details also the children who included more facts?). To achieve these goals we adopted coding schemes, measures and analysis procedures routinely used in the AM literature (AM coding; narrative length and breadth measures), but also introduced a new coding scheme to examine children’s inclusion of semantic facts in their narratives (i.e., facts about animals and animal-related science facts). A secondary and exploratory goal was to determine whether different patterns are observed for different types of experiences. For one event narrative, children described their favorite animal visit (child-selected event; high self-relevance) and for the other event narrative children described an animal visit selected by the experimenter. For various methodological reasons we did not directly or quantitatively compare these two types of events in analyses. However, we can discuss the pattern of findings for each event type and discuss whether patterns differed, while being mindful that self-relevance alone may not fully explain any pattern differences (see limitations below). The present work complement and extend past AM development research. Further, our findings can be useful for staff in informal learning institutions like science centers and zoos, who support children’s education and promote curiosity and excitement about science.

Examination of the types of AM categories (event details) children provide in our study is consistent with past work and can be useful to museum and zoo educators. For example, examination of Table 4 shows that children often included event details that fell into the “what-act” category, similar to past work with autobiographical events from further in the past (Bauer et al., 2007). It is possible that children include more details pertaining to actions because they attend to those features of events. This is consistent with DeMarie (2001) who found that young children often chose to photograph actions when provided with cameras during a field trip to a zoo. Further, our study adds to knowledge about the number and types of animal-related facts that were retained and spontaneously included when children were asked to describe particular events at the zoo (visits to animal exhibits). We found that children included a relatively high number of fact-like details in their narratives, but there was more representation of some fact categories than others as can be seen in the descriptive information provided in Tables 5, 7. Previous studies have found that different age groups tend to focus on different things during field trips to informal learning environments (see Farrar and Goodman, 1992; Birney, 1995; DeMarie, 2001) which has implications for what they will learn from these events and how school programs and field trip programs can be developed based on this knowledge. Educators may also be interested to know that individual differences in narrative breadth was not correlated with individual differences in the overall number of semantic facts. In other words, it is not the case that the children who provided more complete accounts of the events (included more different types of AM categories) were also the children who included more facts in their narratives.

Our findings of age-related improvements in narrative length (for both event types) are consistent with past research that also find that older children provide longer memory narratives than younger children (e.g., Habermas et al., 2010; Bauer and Larkina, 2019; see also Bauer et al., 2019). It is interesting that the 4- to 5- and 6- to 7-year-old groups did not differ in narrative length for the child-selected event, but 6- to 7-year-olds provided longer narratives than 4- to 5-year-olds for the experimenter-selected event. Thus, it is possible that self-relevance increased how much the youngest children wanted to talk about their experiences for this child-selected (favorite animal) event.

In addition to the amount of talk, we also measured the completeness of children’s memory narratives. Given age-related differences in narrative length, we conducted analyses on the narrative breadth measure both with and without controlling for length, an approach used previously (e.g., Bauer et al., 2017). This is important because focusing on only one or the other limits our ability to see the full pattern. For the child-selected event narrative, we found that older children provided more complete narratives (included more different types of AM details; narrative breadth score) than the youngest age group. Once we considered length of narratives, the age-related differences in narrative breadth for the child-selected event narrative disappeared. For the experimenter-selected event narrative, there were age-related improvements (whether or not we controlled for different factors like length). Age-related improvements in narrative breadth scores have been found in several past AM studies (e.g., Bauer and Larkina, 2014, 2019), but not all studies (e.g., Van Abbema and Bauer, 2005). Our finding that 4- to 5- and 6- to 7-year-olds did not differ in narrative length but did show differences in narrative breadth for the child-selected (favorite animal) event narrative is important to note. It suggests that even though these two age groups talked similar amounts, and thus had similar opportunities to provide details in their narratives, they still differed in the number of traditional event detail categories (who, what, where, etc.) that were represented in their narrative (at least when length was not controlled). This particular finding is consistent with Bauer and Larkina (2014) who found that age groups did not differ in their talkativeness, but 5- and 6-year-olds scored lower than 8- and 9-year-olds for narrative breadth.

The present study’s findings on narrative breadth extends past work by comparing general patterns for the two event types. We showed that there were minimal age-related differences in narrative breadth for our self-relevant event (oldest age group only scored higher than younger groups without covariates in analysis; there were no age group differences once we accounted for covariates), but robust age-related differences for the less self-relevant event (age group differences found between all 3 age groups without covariates; age group differences between youngest and oldest groups remained even with all relevant covariates). This pattern of findings is reminiscent of Pathman et al. (2011) because they found that age-related differences in recognition memory accuracy were minimized for a condition which involved high personal involvement compared to a condition that was designed to be less self-relevant. These patterns add to evidence that increasing self-relevance and ownership can boost children’s memory (e.g., Turk et al., 2008; Cunningham et al., 2013, 2014, 2018) and affect adults’ memory accuracy, content, or elaborative processing (see Rogers et al., 1977; Barney, 2007).

Our study is useful for staff at informal learning institutions (and other education settings) because their exhibits and experiences cannot often be tailored to narrow age ranges. Although exploratory, our results suggest that when an event is less self-relevant, there may be larger age gaps in what children include in their AM narratives. It is useful for educators to know that increasing self-relevance or personal involvement may help younger children recall as many types of AM event details as older children. This is also consistent with recommendations from a qualitative study by Wolins et al. (1992) in which they asked children why certain field trips stood out and led researchers to recommend that educators “allow children opportunities for choice, for ways to personalize the experience” (p. 26).

Unlike our findings for AM coding, self-relevance did not seem to boost children’s inclusion of facts in their narratives. We found, for both the child-selected and experimenter-selected events, that older children’s narratives contained more facts (overall semantic score) compared to younger children. However, when we controlled for narrative length, age groups differences were no longer apparent. Thus, our results suggest that although self-relevance impacts the amount of autobiographical/episodic event details, it may not impact the total number of facts children choose to discuss. We also see that the particular fact categories driving age differences (before controlling for covariates) showed both similarities and differences across the two event narratives. For both event types, age-group differences were apparent for the TF and AF categories. Thus, compared to younger children, older children included more facts that required remembering unobservable concepts and semantic details and required remembering names of subgroups for which particular information applied. In other words, for both event narratives older children included, arguably, more challenging semantic information than younger children – more challenging because this information is unlikely to be produced or reconstructed based on memory for event details. Age group differences for target facts is consistent with age-related improvements in children’s ability to learn and perceive conceptual hierarchies in early to middle childhood (e.g., Schaeffer et al., 1971; Whitney and Kunen, 1983) and remember specific labels in generic statements (Gülgöz and Gelman, 2015). Age group differences for AFs is consistent with improvements across childhood in the ability to associate knowledge with existing mental concepts during the learning process (see discussions Gelman and Brenneman, 2004). At the same time, two fact categories did not show similar effects for the two types of event narratives. Older children provided more CFs (e.g., visually observable information) than younger children for the child-selected event narratives, but this age difference was not there for the experimenter-selected event narrative. For the experimenter-selected event narrative, older children included more facts having to do with an animal’s behavior in their narratives, compared to younger children. We do not want to make strong claims about these differences between the two event types. Overall, however, it is useful for museum educators to know what types of facts are included when children are asked to recall their experience visiting an exhibit and the things they learned (as a reminder, our autobiographical interview included the standard questions used in past research, plus an additional sub-question question in which children were asked about the cool/neat things they learned). Further, it is useful to know for what types of fact categories younger and older children are showing similar levels of learning and for what types of fact categories younger children are trailing behind older children. Future studies are needed to see if our findings about the different fact categories represented would be replicated in other zoo or science centers that contain animal exhibits.

Our goals were to examine children’s memory and learning following engaging experiences at a local zoo. By examining both autobiographical event details and semantic details included in response to open-ended questions we determined what types of details were recalled and whether there were age group differences in their recall. Unlike previous AM coding studies that did not distinguish past event details from fact-like details (they were both included in AM coding), we additionally determined different categories of semantic facts that were represented in children’s autobiographical narratives. These facts could have been remembered because they heard a zoo staff member providing that information, and the child was able to then recite that information in their narrative. These facts could also have been generated by the child based on their own observations at the zoo. For example, it is possible that a child stated that giraffes have dark tongues because they remembered that the particular giraffe they visited had a black tongue and generalized this information to all giraffes. Of course, the latter example is more likely to have occurred for some semantic codes in our study (e.g., CFs) and the former is more likely to have occurred for other semantic codes (e.g., AFs). However, this is an empirical question and future studies could observe or record children’s experiences during the animal visit to determine the various sources of new semantic information children later incorporate into their narratives.

Observing and/or recording children’s experiences and conversations during museum visits have been successfully used in several past studies (e.g., Benjamin et al., 2010; Cox-Peterson et al., 2003; Palmquist and Crowley, 2007; Rigney and Callanan, 2011), two of which are especially relevant to the present work because they discussed both autobiographical/episodic memory and learning. Jant et al. (2014) recorded children and their parents during their visit of two museum exhibits and, for a subset of participants, also obtained recordings of conversations parents had with their children about their memories for the museum experiences. These researchers observed that the conversations consisted of both episodic details and semantic details. Imuta et al. (2018) built on this observation in their study. Researchers interviewed 5- and 6-year-old children after a science lesson that they experienced in either a field trip context or in a classroom context (between-subjects design). They asked children what they remembered about each experience and coded children’s responses into two categories: autobiographical information (i.e., info about what happened during that event) and scientific information (i.e., information about something they learned). Researchers found that for autobiographical information, but not scientific information, children recalled more in the fieldtrip context than the classroom context after a delay of 1–2 days. Although the goal of the present study was not to compare different learning contexts, our study extends the work of Imuta and colleagues by examining multiple sub-categories of details within the autobiographical and semantic information categories. Imuta and colleagues found that the amount of autobiographical information children recalled was predictive of the amount of scientific information they recalled. This is in contrast to our study because we did not find that individual differences in AM narrative breadth was correlated with individual differences in the overall semantic score, at least when we controlled for narrative length. As far as we can tell, Imuta and colleagues did not control for the amount of talk in their analyses, which could account for the different findings. Future studies could help to clarify whether or not children who provide more autobiographical details also provide more semantic details. Future work would also benefit from examination of other types of individual differences that could impact children’s learning and memory.

We did not find gender differences in any of our AM measures (length and breadth) for either event. We also found no gender differences for the semantic measure for the child-selected event. However, for the experimenter-selected event we found that for the 8- to 10-year-old age group boys included more semantic information in their narratives than girls. Given that this gender difference is isolated to only one age group and only to one of the two event narratives, this effect should be interpreted cautiously, and future studies are needed to determine if this effect would be replicated. If this effect is replicated, then additional research would help to explain why there may be an effect of gender on our semantic memory measure. For example, did older boys ask more questions from zoo staff and thus hear more semantic information during the experimenter-selected event? Imuta and colleagues did not examine gender effects in their study. However, advantages for boys have been found in other studies about science learning. For example, Crowley et al. (2001b) examined parent-child conversations at a science exhibit and found that parents provided more explanations when speaking with boys compared to girls. Further, Tenenbaum et al. (2005) showed mothers playing with magnets with their children engaged in more science talk with boys compared to girls (and with older children compared to younger children). Still, other museum studies have found no systematic gender differences (Crowley et al., 2001a; Benjamin et al., 2010; Haden et al., 2014; Jant et al., 2014). Thus, there are mixed findings about gender differences in relation to science learning. Studies are also mixed in terms of gender differences in AM narratives such that some have found girls provide longer and/or more complete AM narratives than boys (e.g., Buckner and Fivush, 1998; Bauer et al., 2007), whereas other studies have not found gender differences (see review Grysman and Hudson, 2013). We also did not find gender differences in our AM narratives. Our original goal was not to examine gender differences, and so these results should be interpreted cautiously. Future studies on children’s experiences at informal learning environments that are designed to examine both gender and age group differences, but also individual differences in other domains, are needed and would help to determine ways to optimize memory and learning outcomes.

Several caveats and limitations about the present study should be noted. First, our data were based on open-ended questions, paralleling past AM studies. We examined the number and types of semantic facts children included in their narratives spontaneously. It is possible that children would have recalled more information with more specific cueing (e.g., “What did you learn about a rhino’s horn tissue?”). As such, future work could incorporate both open-ended narrative questions and direct fact-based questions. Second, we did not video-record individual children’s visits to exhibits throughout the week and thus do not know exactly what was seen and heard by children. Thus, our study cannot tell us about the proportion of total *possible* event details and facts that were recalled and how much of that was accurate. Such a study would be laborious (coding videos for all possible episodic and semantic details to determine what exactly was experienced by each child), but is a needed extension of the present work.

Another planned limitation was that the order in which we elicited the narratives for the two events were always the same: child-selected event narrative was obtained before the experimenter-selected event narrative. This was necessary in our study for several reasons, including knowing that time limitations would not allow us to test both event types in all children, that the experimenter-selected event was constrained to particular experiences, and importantly we needed the child-selected event narrative to occur first so that the experimenter-selected event would not be about the same event. Thus, we planned not to include both event types in the same analysis. However, this meant that in addition to the two events differing in the amount of self-relevance, it is also the case that describing the experimenter-selected event could have been more taxing for children because it was always later in the interview. Future studies could extend this work by making the primary goal of the study directly comparing event types based on these findings. Future studies could also examine why there may have been an advantage for the child-selected event. In the present work we do not know whether children chose a particular animal as their favorite because this preference existed prior to attending zoo camp, and if this preference caused children to have increased attention to that particular animal visit. Studies have found a link between curiosity and learning (see Gruber et al., 2014; Oudeyer et al., 2016). On the other hand, it is possible that children established a particular animal as their favorite after seeing that exhibit. A future adaptation of the present work could involve interviewing children before attending zoo camp to determine how pre-existing preferences may influence later memory and learning, similar to a study that examined children’s knowledge about what usually happens during visits to the zoo before and after a zoo experience (DeMarie et al., 2000).

Finally, one of the features of the present study was that it involved an extended set of experiences over a 5-day span. However, this meant that children had multiple opportunities for conversations with others about their experiences during this time period, before our test session on the last day of camp. For instance, it is likely that children spoke about their camp experiences, including animal visits, with parents at home. Leichtman et al. (2017) found that parents’ conversational styles influenced the amount of information children contributed during the conversation with parents, and this in turn was correlated with how much they remembered in an interview with researchers 6 days later. In the present work, we did not examine how conversations with peers during the camp, or at home with their parents, influenced their retention of event details and fact knowledge, but this would be an interesting line of future work.

Field trips or trips to informal learning institutions not only act as a naturalistic learning setting but have also exhibited a strong potential for improvement of cognitive development, critical thinking skills and as motivators for advanced learning (Hurley, 2006; Greene et al., 2014). Our work echoes these findings and demonstrates that informal learning institutes are an engaging method for children to learn and recall information. Educators can captivate children’s attention by actively asking children questions to help them attend to specific details rather than requiring them to passively listen to information. This may be especially helpful for younger age-groups who displayed a lower number of autobiographical and semantic recall than older age groups for certain types of events. Encouraging children and parents to discuss the event and what children learned about can also prove to be helpful for recall of scientific information (Leichtman et al., 2017).

To conclude, using a controlled naturalistic study, we examined children’s memory for event details and the retention of fact knowledge after a week-long summer camp at a local zoo. In addition to extending previous studies on AM, we determined the types of science-related facts children included in their AM narratives and how that changed across early to middle childhood. We also discuss the various ways future studies can extend our results. We hope the present line of work along with the existing literature (e.g., Birney, 1995) can be useful for science educators and informal learning environments to promote children’s memory and learning outcomes.

## Data Availability Statement

The raw data supporting the conclusions of this article will be made available by the authors, without undue reservation.

## Ethics Statement

The studies involving human participants were reviewed and approved by Research Ethics Board at York University. Written informed consent to participate in this study was provided by the participants’ legal guardian/next of kin.

## Author Contributions

TP conceptualized, designed, supervised the research, and conducted the analysis. TK, PP, and TP contributed to the data collection. TK, PP, and GF contributed to the narrative coding, reliability analysis, and data entering/processing. All authors contributed in different ways to writing and revising the manuscript. TK and GF used portions of the data for their undergraduate honors thesis projects. All authors contributed to the article and approved the submitted version.

## Conflict of Interest

The authors declare that the research was conducted in the absence of any commercial or financial relationships that could be construed as a potential conflict of interest.
